# 2-Amino-4,6-dimethyl­pyridinium chloride dihydrate

**DOI:** 10.1107/S1600536811035100

**Published:** 2011-09-14

**Authors:** Mohammad T. M. Al-Dajani, Jamal Talaat, Abdusalam Salhin, Madhukar Hemamalini, Hoong-Kun Fun

**Affiliations:** a School of Pharmaceutical Sciences, Universiti Sains Malaysia, 11800 USM, Penang, Malaysia; b Virginia Commonwealth University, Chemistry School, USA; c School of Chemical Sciences, Universiti Sains Malaysia, 11800 USM, Penang, Malaysia; d X-ray Crystallography Unit, School of Physics, Universiti Sains Malaysia, 11800 USM, Penang, Malaysia

## Abstract

In the title hydrated mol­ecular salt, C_7_H_11_N_2_^+^·Cl^−^·2H_2_O, the pyridine N atom of the 2-amino-4,6-dimethyl­pyridine mol­ecule is protonated. The cation is essentially planar, with a maximum deviation of 0.006 (2) Å. In the crystal, the components are linked by N—H⋯O, N—H⋯Cl and O—H⋯Cl hydrogen bonds, thereby forming sheets lying parallel to (100). The crystal structure is further stabilized by aromatic π–π stacking inter­actions between the pyridinium rings [centroid–centroid distance = 3.4789 (9) Å].

## Related literature

For details of 2-amino­pyridine and its derivatives, see: Katritzky *et al.* (1996 ▶). For pyridine derivatives as templating agents, see: Matsumoto (2003 ▶); Desiraju (2001 ▶); Bond & Parsons (2002 ▶).
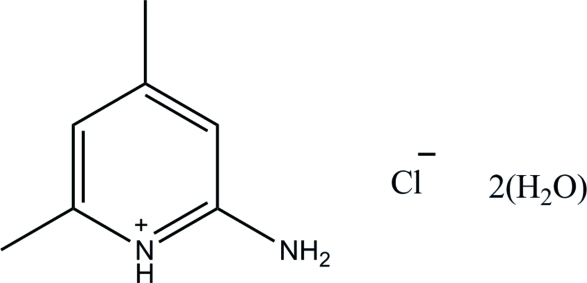


## Experimental

### 

#### Crystal data

C_7_H_11_N_2_^+^·Cl^−^·2H_2_O
*M* *_r_* = 194.66
Monoclinic, 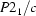

*a* = 7.5811 (6) Å
*b* = 13.8149 (11) Å
*c* = 10.6657 (8) Å
β = 109.261 (2)°
*V* = 1054.52 (14) Å^3^
*Z* = 4
Mo *K*α radiation
μ = 0.33 mm^−1^
*T* = 296 K
0.44 × 0.18 × 0.05 mm

#### Data collection

Bruker APEXII DUO CCD diffractometer
Absorption correction: multi-scan (*SADABS*; Bruker, 2009 ▶) *T*_min_ = 0.868, *T*_max_ = 0.982
16733 measured reflections
3109 independent reflections
2020 reflections with *I* > 2σ(*I*)
*R*_int_ = 0.029

#### Refinement

*R*[*F* ^2^ > 2σ(*F* ^2^)] = 0.038
*wR*(*F* ^2^) = 0.126
*S* = 1.04
3109 reflections
127 parameters
H atoms treated by a mixture of independent and constrained refinement
Δρ_max_ = 0.16 e Å^−3^
Δρ_min_ = −0.25 e Å^−3^

### 

Data collection: *APEX2* (Bruker, 2009 ▶); cell refinement: *SAINT* (Bruker, 2009 ▶); data reduction: *SAINT*; program(s) used to solve structure: *SHELXTL* (Sheldrick, 2008 ▶); program(s) used to refine structure: *SHELXTL*; molecular graphics: *SHELXTL*; software used to prepare material for publication: *SHELXTL* and *PLATON* (Spek, 2009 ▶).

## Supplementary Material

Crystal structure: contains datablock(s) global, I. DOI: 10.1107/S1600536811035100/hb6390sup1.cif

Structure factors: contains datablock(s) I. DOI: 10.1107/S1600536811035100/hb6390Isup2.hkl

Supplementary material file. DOI: 10.1107/S1600536811035100/hb6390Isup3.cml

Additional supplementary materials:  crystallographic information; 3D view; checkCIF report

## Figures and Tables

**Table 1:** Hydrogen-bond geometry (Å, °)

*D*—H⋯*A*	*D*—H	H⋯*A*	*D*⋯*A*	*D*—H⋯*A*
O2*W*—H1*WA*⋯Cl1	0.84 (3)	2.37 (3)	3.2076 (17)	179 (3)
O2*W*—H2*WA*⋯Cl1^i^	0.83 (3)	2.39 (3)	3.1689 (16)	157 (3)
O1*W*—H1*WB*⋯Cl1^ii^	0.84 (3)	2.36 (3)	3.2019 (15)	179 (3)
O1*W*—H2*WB*⋯Cl1^iii^	0.79 (2)	2.41 (2)	3.1916 (16)	172.4 (18)
N1—H1*N*1⋯O2*W*	0.86	1.93	2.7852 (18)	174
N2—H2*N*1⋯Cl1^i^	0.86	2.53	3.3177 (12)	153
N2—H2*N*2⋯O1*W*^iv^	0.86	2.00	2.8543 (17)	175

Symmetry codes: (i) 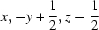; (ii) 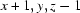; (iii) 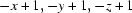; (iv) 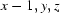.

**Fig. 1.**
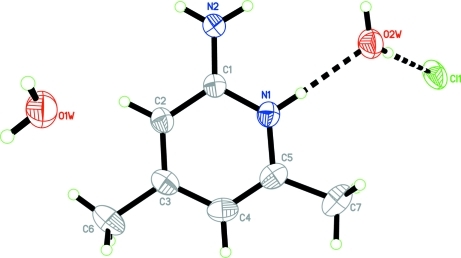
The asymmetric unit of the title compound, showing 50% probability displacement ellipsoids. The hydrogen bonds are shown by dashed lines.

**Fig. 2.**
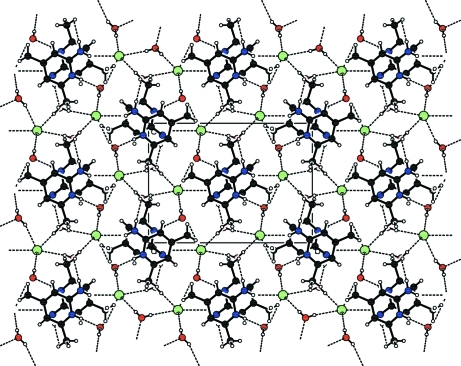
The crystal packing of title compound, (I), looking down the a-axis.

## supplementary crystallographic information

### Comment

2-Aminopyridine and its derivatives play an important role in heterocyclic chemistry (Katritzky *et al.*, 1996).  The use of pyridine derivatives as templating agents for the self-assembly of organic–inorganic supramolecular materials has been widely studied (Matsumoto, 2003; Desiraju, 2001; Bond & Parsons, 2002).  In order to further study hydrogen bonding interactions in these systems, the synthesis and structure of the title salt (I) is presented here.

The asymmetric unit of the title compound, (I), contains a 2-amino-4,6- dimethylpyridinium cation, a chloride anion and two water molecules as shown in Fig. 1. The cation (N1/C1–C5) is essentially planar, with a maximum deviation of 0.006 (2) Å for atom C5.  In the 2-amino-4,6- dimethylpyridinium cation, a wider than normal angle [123.22 (12)°] is subtended at the protonated N1 atom.

In the crystal structure, (Fig. 2), the ion pais and water molecules are linked *via* O2W—H1WA···Cl1, O2W—H2WA···Cl1, O1W—H1WA···Cl1, O1W—H2WA···Cl1, N1—H1N1···O2W, N2—H2N1···Cl1 and N2—H2N2···O1W hydrogen bonds (Table 1) forming two-dimensional networks parallel to the (100)-plane.  The crystal structure is further stabilized by π–π- interactions between the pyridinium (Cg1; N1/C1–C5) rings [Cg1···Cg1 = 3.4789 (9) Å; 1-x, 1-y, 1-z].

### Experimental

In a round bottom flask, 25ml of tetrahydrofuran (THF) was mixed with 2-amino-4,6-dimethylpyridine (0.01 mol, 1.3 g) with stirring.  Drops of benzoyl chloride (0.01 mol, 1.0 g) dissolved in THF was then added. The reaction mixture was refluxed for 30 min.  The precipitate formed was washed with THF. The precipitate was then dissolved in methanol at room temperature. After few days, colorless plates of (I) were formed by slow evaporation.

### Refinement

Atoms H1WA, H2WA, H1WB and H2WB were located from a difference Fourier maps and refined freely [O–H = 0.78 (2)–0.84 (3) Å]. The remaining H atoms were positioned geometrically [N–H = 0.86 Å and C–H = 0.96 Å] and were refined using a riding model, with *U*_iso_(H) = 1.2 or 1.5 *U*_eq_(C).  A rotating group model was applied to the methyl groups.

### Crystal data

**Table:**

C_7_H_11_N_2_^+^·Cl^−^·2H_2_O	*F*(000) = 416
*M**_r_* = 194.66	*D*_x_ = 1.226 Mg m^−^^3^
Monoclinic, *P*2_1_/*c*	Mo *K*α radiation, λ = 0.71073 Å
Hall symbol: -P 2ybc	Cell parameters from 3933 reflections
*a* = 7.5811 (6) Å	θ = 2.5–26.6°
*b* = 13.8149 (11) Å	µ = 0.33 mm^−^^1^
*c* = 10.6657 (8) Å	*T* = 296 K
β = 109.261 (2)°	Plate, colourless
*V* = 1054.52 (14) Å^3^	0.44 × 0.18 × 0.05 mm
*Z* = 4	

### Data collection

**Table:**

Bruker APEXII DUO CCD diffractometer	3109 independent reflections
Radiation source: fine-focus sealed tube	2020 reflections with *I* > 2σ(*I*)
graphite	*R*_int_ = 0.029
φ and ω scans	θ_max_ = 30.2°, θ_min_ = 2.5°
Absorption correction: multi-scan (*SADABS*; Bruker, 2009)	*h* = −10→10
*T*_min_ = 0.868, *T*_max_ = 0.982	*k* = −19→19
16733 measured reflections	*l* = −15→15

### Refinement

**Table:**

Refinement on *F*^2^	Primary atom site location: structure-invariant direct methods
Least-squares matrix: full	Secondary atom site location: difference Fourier map
*R*[*F*^2^ > 2σ(*F*^2^)] = 0.038	Hydrogen site location: inferred from neighbouring sites
*wR*(*F*^2^) = 0.126	H atoms treated by a mixture of independent and constrained refinement
*S* = 1.04	*w* = 1/[σ^2^(*F*_o_^2^) + (0.0598*P*)^2^ + 0.1086*P*] where *P* = (*F*_o_^2^ + 2*F*_c_^2^)/3
3109 reflections	(Δ/σ)_max_ < 0.001
127 parameters	Δρ_max_ = 0.16 e Å^−^^3^
0 restraints	Δρ_min_ = −0.25 e Å^−^^3^

### Special details

**Table:**

Geometry. All esds (except the esd in the dihedral angle between two l.s. planes) are estimated using the full covariance matrix. The cell esds are taken into account individually in the estimation of esds in distances, angles and torsion angles; correlations between esds in cell parameters are only used when they are defined by crystal symmetry. An approximate (isotropic) treatment of cell esds is used for estimating esds involving l.s. planes.
Refinement. Refinement of F^2^ against ALL reflections. The weighted R-factor wR and goodness of fit S are based on F^2^, conventional R-factors R are based on F, with F set to zero for negative F^2^. The threshold expression of F^2^ > 2sigma(F^2^) is used only for calculating R-factors(gt) etc. and is not relevant to the choice of reflections for refinement. R-factors based on F^2^ are statistically about twice as large as those based on F, and R- factors based on ALL data will be even larger.

### Fractional atomic coordinates and isotropic or equivalent isotropic displacement parameters (Å^2^)

**Table:**

	*x*	*y*	*z*	*U*_iso_*/*U*_eq_	
O2W	0.2219 (2)	0.29451 (10)	0.70681 (15)	0.0690 (3)	
H1WA	0.175 (3)	0.3025 (18)	0.767 (3)	0.114 (9)*	
H2WA	0.167 (3)	0.2532 (18)	0.652 (3)	0.099 (8)*	
O1W	0.9019 (2)	0.45839 (11)	0.12748 (12)	0.0714 (4)	
H1WB	0.940 (4)	0.422 (2)	0.079 (3)	0.134 (11)*	
H2WB	0.907 (3)	0.5121 (17)	0.105 (2)	0.081 (7)*	
Cl1	0.04165 (8)	0.32061 (3)	0.93781 (4)	0.0800 (2)	
C4	0.4433 (2)	0.61916 (12)	0.62946 (16)	0.0601 (4)	
H4A	0.5228	0.6649	0.6833	0.072*	
C5	0.4204 (2)	0.53267 (12)	0.68093 (15)	0.0551 (4)	
C6	0.3769 (3)	0.73643 (12)	0.4383 (2)	0.0802 (5)	
H6A	0.3130	0.7366	0.3442	0.120*	
H6B	0.5080	0.7468	0.4555	0.120*	
H6C	0.3281	0.7872	0.4788	0.120*	
C7	0.5173 (3)	0.50077 (17)	0.82075 (17)	0.0811 (5)	
H7A	0.5860	0.4424	0.8205	0.122*	
H7B	0.4264	0.4888	0.8638	0.122*	
H7C	0.6017	0.5505	0.8677	0.122*	
C1	0.20566 (17)	0.48350 (9)	0.47123 (12)	0.0431 (3)	
C3	0.3482 (2)	0.64080 (10)	0.49498 (16)	0.0543 (3)	
C2	0.2309 (2)	0.57277 (10)	0.41758 (14)	0.0490 (3)	
H2A	0.1673	0.5860	0.3286	0.059*	
N1	0.30065 (15)	0.46692 (8)	0.60065 (11)	0.0474 (3)	
H1N1	0.2851	0.4123	0.6343	0.057*	
N2	0.09263 (17)	0.41503 (8)	0.40042 (11)	0.0521 (3)	
H2N1	0.0809	0.3611	0.4373	0.063*	
H2N2	0.0313	0.4247	0.3178	0.063*	

### Atomic displacement parameters (Å^2^)

**Table:**

	*U*^11^	*U*^22^	*U*^33^	*U*^12^	*U*^13^	*U*^23^
O2W	0.0867 (9)	0.0645 (7)	0.0593 (8)	0.0020 (6)	0.0287 (7)	0.0074 (6)
O1W	0.1032 (10)	0.0577 (7)	0.0561 (7)	0.0001 (7)	0.0301 (7)	0.0029 (5)
Cl1	0.1373 (5)	0.0461 (2)	0.0693 (3)	0.0112 (2)	0.0511 (3)	0.00246 (16)
C4	0.0540 (8)	0.0599 (9)	0.0679 (10)	−0.0043 (7)	0.0221 (7)	−0.0188 (7)
C5	0.0485 (7)	0.0656 (9)	0.0511 (8)	0.0060 (7)	0.0163 (6)	−0.0097 (6)
C6	0.0912 (12)	0.0466 (9)	0.1132 (16)	−0.0066 (8)	0.0478 (11)	0.0025 (9)
C7	0.0712 (11)	0.1110 (16)	0.0514 (10)	0.0050 (11)	0.0073 (8)	−0.0053 (9)
C1	0.0456 (7)	0.0424 (6)	0.0445 (7)	0.0055 (5)	0.0192 (6)	0.0002 (5)
C3	0.0556 (8)	0.0432 (7)	0.0729 (10)	0.0015 (6)	0.0332 (7)	−0.0040 (6)
C2	0.0560 (8)	0.0438 (7)	0.0522 (8)	0.0054 (6)	0.0247 (6)	0.0031 (5)
N1	0.0502 (6)	0.0481 (6)	0.0454 (6)	0.0066 (5)	0.0179 (5)	0.0030 (4)
N2	0.0621 (7)	0.0436 (6)	0.0478 (6)	−0.0038 (5)	0.0143 (5)	0.0023 (5)

### Geometric parameters (Å, °)

**Table:**

O2W—H1WA	0.84 (3)	C6—H6C	0.9600
O2W—H2WA	0.83 (3)	C7—H7A	0.9600
O1W—H1WB	0.84 (3)	C7—H7B	0.9600
O1W—H2WB	0.78 (2)	C7—H7C	0.9600
C4—C5	1.350 (2)	C1—N2	1.3314 (17)
C4—C3	1.409 (2)	C1—N1	1.3493 (17)
C4—H4A	0.9300	C1—C2	1.3989 (18)
C5—N1	1.3673 (18)	C3—C2	1.368 (2)
C5—C7	1.495 (2)	C2—H2A	0.9300
C6—C3	1.498 (2)	N1—H1N1	0.8600
C6—H6A	0.9600	N2—H2N1	0.8600
C6—H6B	0.9600	N2—H2N2	0.8600
			
H1WA—O2W—H2WA	113 (2)	H7A—C7—H7C	109.5
H1WB—O1W—H2WB	108 (2)	H7B—C7—H7C	109.5
C5—C4—C3	120.70 (14)	N2—C1—N1	119.09 (12)
C5—C4—H4A	119.7	N2—C1—C2	122.92 (12)
C3—C4—H4A	119.7	N1—C1—C2	117.99 (12)
C4—C5—N1	118.78 (14)	C2—C3—C4	118.76 (14)
C4—C5—C7	125.43 (15)	C2—C3—C6	120.95 (15)
N1—C5—C7	115.79 (15)	C4—C3—C6	120.28 (15)
C3—C6—H6A	109.5	C3—C2—C1	120.54 (13)
C3—C6—H6B	109.5	C3—C2—H2A	119.7
H6A—C6—H6B	109.5	C1—C2—H2A	119.7
C3—C6—H6C	109.5	C1—N1—C5	123.22 (12)
H6A—C6—H6C	109.5	C1—N1—H1N1	118.4
H6B—C6—H6C	109.5	C5—N1—H1N1	118.4
C5—C7—H7A	109.5	C1—N2—H2N1	120.0
C5—C7—H7B	109.5	C1—N2—H2N2	120.0
H7A—C7—H7B	109.5	H2N1—N2—H2N2	120.0
C5—C7—H7C	109.5		
			
C3—C4—C5—N1	−1.0 (2)	N2—C1—C2—C3	179.71 (12)
C3—C4—C5—C7	178.37 (15)	N1—C1—C2—C3	−0.19 (19)
C5—C4—C3—C2	0.5 (2)	N2—C1—N1—C5	179.69 (12)
C5—C4—C3—C6	−179.05 (14)	C2—C1—N1—C5	−0.40 (18)
C4—C3—C2—C1	0.2 (2)	C4—C5—N1—C1	1.01 (19)
C6—C3—C2—C1	179.67 (13)	C7—C5—N1—C1	−178.43 (13)

### Hydrogen-bond geometry (Å, °)

**Table:**

*D*—H···*A*	*D*—H	H···*A*	*D*···*A*	*D*—H···*A*
O2W—H1WA···Cl1	0.84 (3)	2.37 (3)	3.2076 (17)	179 (3)
O2W—H2WA···Cl1^i^	0.83 (3)	2.39 (3)	3.1689 (16)	157 (3)
O1W—H1WB···Cl1^ii^	0.84 (3)	2.36 (3)	3.2019 (15)	179 (3)
O1W—H2WB···Cl1^iii^	0.79 (2)	2.41 (2)	3.1916 (16)	172.4 (18)
N1—H1N1···O2W	0.86	1.93	2.7852 (18)	174
N2—H2N1···Cl1^i^	0.86	2.53	3.3177 (12)	153
N2—H2N2···O1W^iv^	0.86	2.00	2.8543 (17)	175

Symmetry codes: (i) *x*, −*y*+1/2, *z*−1/2; (ii) *x*+1, *y*, *z*−1; (iii) −*x*+1, −*y*+1, −*z*+1; (iv) *x*−1, *y*, *z*.
